# Phospholipase C-Related Catalytically Inactive Protein Participates in the Autophagic Elimination of *Staphylococcus aureus* Infecting Mouse Embryonic Fibroblasts

**DOI:** 10.1371/journal.pone.0098285

**Published:** 2014-05-27

**Authors:** Kae Harada-Hada, Kana Harada, Fuminori Kato, Junzo Hisatsune, Isei Tanida, Michinaga Ogawa, Satoshi Asano, Motoyuki Sugai, Masato Hirata, Takashi Kanematsu

**Affiliations:** 1 Department of Cellular and Molecular Pharmacology, Institute of Biomedical and Health Sciences, Hiroshima University, Hiroshima, Japan; 2 Department of Bacteriology, Institute of Biomedical and Health Sciences, Hiroshima University, Hiroshima, Japan; 3 Department of Biochemistry and Cell Biology, National Institute of Infectious Diseases, Tokyo, Japan; 4 Department of Bacteriology I, National Institute of Infectious Diseases, Tokyo, Japan; 5 Laboratory of Molecular and Cellular Biochemistry, Faculty of Dental Science, Kyushu University, Fukuoka, Japan; The Tokyo Metropolitan Institute Medical Science, Japan

## Abstract

Autophagy is an intrinsic host defense system that recognizes and eliminates invading bacterial pathogens. We have identified microtubule-associated protein 1 light chain 3 (LC3), a hallmark of autophagy, as a binding partner of phospholipase C-related catalytically inactive protein (PRIP) that was originally identified as an inositol trisphosphate-binding protein. Here, we investigated the involvement of PRIP in the autophagic elimination of *Staphylococcus aureus* in infected mouse embryonic fibroblasts (MEFs). We observed significantly more LC3-positive autophagosome-like vacuoles enclosing an increased number of *S. aureus* cells in *PRIP*-deficient MEFs than control MEFs, 3 h and 4.5 h post infection, suggesting that *S. aureus* proliferates in LC3-positive autophagosome-like vacuoles in *PRIP*-deficient MEFs. We performed autophagic flux analysis using an mRFP-GFP-tagged LC3 plasmid and found that autophagosome maturation is significantly inhibited in *PRIP*-deficient MEFs. Furthermore, acidification of autophagosomes was significantly inhibited in *PRIP*-deficient MEFs compared to the wild-type MEFs, as determined by LysoTracker staining and time-lapse image analysis performed using mRFP-GFP-tagged LC3. Taken together, our data show that PRIP is required for the fusion of *S. aureus*-containing autophagosome-like vacuoles with lysosomes, indicating that PRIP is a novel modulator in the regulation of the innate immune system in non-professional phagocytic host cells.

## Introduction

Autophagy, an evolutionarily conserved intracellular catabolic pathway in eukaryotic cells, delivers intracellular materials, such as damaged cytosolic components, into the lysosomes for degradation. Autophagy also plays an important role in eliminating invading pathogens by targeting them to the lysosome [1]. We recently reported that phospholipase C (PLC)-related catalytically inactive protein (PRIP) is a modulator for canonical autophagy [2]. However, it is unknown whether PRIP is involved in the autophagy-mediated clearance of intracellular pathogens.

In the autophagy pathway, a part of the cytoplasm is sequestered by autophagosomes, which in mammals are double-membrane vacuoles characterized by the presence of specific structures containing microtubule-associated protein 1 light chain 3 (LC3), a homologue of yeast autophagy-related protein 8 (Atg8) [3], [4]. The multiple steps of autophagy generally consist of the formation of a phagophore, which is the membrane precursor of the autophagosome; the elongation and closure of the membrane; and the maturation of autophagosomes by fusion with lysosomes, resulting in the formation of autolysosomes, thus acquiring an acidic compartment for degradation [5].

Xenophagy, an autophagic pathway triggered by microbial infection to combat intracellular pathogens, is a host defense mechanism that serves to restrict bacterial growth and thus the infection of neighboring cells. For some pathogens, however, autophagosomes may be beneficial to the invading microbe in terms of supporting replication and pathogenesis, and thus promoting the pathogen life cycle [6]. *Coxiella burnetti*, *Brucella abortus*, and *Porphyromonas gingivalis*, for example, can reside in the autophagosome and utilize the nutrients sequestered by the vesicle for their growth and proliferation [7].

*Staphylococcus aureus* is a pathogen that causes serious diseases including pneumonia, endocarditis, and osteomyelitis, in addition to wound infection. The proliferation of accessory gene regulator (*agr*)-positive *S. aureus* strains has been reported to be markedly impaired in mouse embryonic fibroblasts (MEFs) deficient of the autophagy protein Atg5, indicating an essential role for the autophagic pathway in *S. aureus* replication [8]. On the other hand, Mauthe and his colleagues recently reported that intracellular *agr*-positive *S. aureus* is sequestered by autophagosome-like vacuoles decorated with WD repeat domain phosphoinositide-interacting protein 1 (WIPI-1), and that, like in the canonical autophagic pathway, these vacuoles become more abundant upon lysosomal inhibition. From these findings, the authors concluded that *S. aureus* is degraded by xenophagy [9]. To date, it remains unclear whether *S. aureus* is eliminated by the autophagic pathway or whether it is sequestered by autophagosomes, where it proliferates and then escapes into the cytoplasm.

PRIP was originally identified as a _D_-*myo*-inositol 1,4,5-trisphosphate-binding protein in rat brain [10], and has a domain organization similar to that of phospholipase-C δ1, but lacks enzyme activity [11]–[13], comprising two isoforms, PRIP1 and PRIP2 [14], [15]. PRIP regulates intracellular inositol 1,4,5-trisphosphate/Ca^2+^ signaling via the pleckstrin homology domain [16], [17]. The functional aspects of PRIP have been characterized using *PRIP1* knockout (*PRIP1*-KO) or *PRIP1* and *PRIP2* double-knockout (*PRIP*-DKO) mice, as well as by studying PRIP-binding partners [18]. To date, we have found that PRIP binds GABA_A_-receptor-associated protein (GABARAP), a mammalian paralog of LC3, and regulates GABARAP-dependent GABA_A_ receptor intracellular trafficking [19], [20]. We have also identified the GABA_A_ receptor β subunit [21], [22], the catalytic subunit of protein phosphatase 1 [23]–[26], protein phosphatase 2A [21], [26], and phospho-Akt/protein kinase B [27] as binding partners of PRIP. These findings led us to conclude that by regulating protein phosphatase 1, protein phosphatase 2A, and phosphorylated Akt, PRIP participates in the phospho-dependent modulation of GABA_A_ receptor function and trafficking, as well as in SNAP-25-phosphoregulated exocytosis [24], [27]–[29].

We have recently demonstrated that PRIP regulates amino acid starvation-induced autophagic flux by binding to LC3 [2]. Therefore, we used MEFs prepared from *PRIP*-DKO mice and wild-type (WT) mice to examine whether PRIP is involved in the elimination of *S. aureus* by the autophagic pathway, and, thus, determined that PRIP is a novel modulator for maintenance of the innate immune system in non-professional phagocytic host cells.

## Materials and Methods

### Plasmids

The expression plasmids pmRFP-LC3 and pmRFP-GFP-LC3 were obtained from Addgene (Cambridge, MA). GFP-PRIP1 was prepared as previously described [16].

### Bacterial strains and growth conditions

*S. aureus* strains MW2 and ATCC 29213 were grown on tryptic soy agar (Becton Dickinson, Franklin Lakes, NJ). A colony from the agar plates was cultured in tryptic soy broth (Becton) at 37°C until the mid-logarithmic phase of growth before being used in the infection assays. *S. aureus* ATCC 29213 was transformed with a modified pS1-GFP plasmid [8] using the Gene Pulser II electroporation system (Bio-Rad, Hercules, CA). A positive clone on a chloramphenicol-containing tryptic soy agar plate was used in the subsequent experiments.

### Eukaryotic cell culture and transfection

The preparation of *PRIP*-DKO MEFs was conducted as previously reported [2], [21]. MEFs stored in liquid nitrogen were grown in Dulbecco's modified Eagle medium (DMEM; Sigma-Aldrich, St. Louis, MO) supplemented with 15% fetal bovine serum (FBS; Gibco/Life Technologies, Carlsbad, CA) and 1% penicillin/streptomycin (Nakalai Tesque Inc., Kyoto, Japan). Cultures were maintained at 37°C in a humidified 5% CO_2_ incubator, as previously described [2]. Plasmids were transfected into MEFs using 4D-Nucleofector and a 4D-Nucleofector X Kit (Lonza, Basel, Switzerland), according to the manufacturer's instructions. Briefly, cells (5×10^5^ cells) were resuspended in 100 µL of 4D-Nucleofector solution and transfected with 2 µg of each plasmid. The transfected cells were seeded onto glass coverslips and allowed to adhere overnight in culture medium (DMEM containing 15% FBS without antibiotics).

### *S. aureus* infection of MEFs

*In vitro* MEF infection was performed as previously described [30], with minor modifications. Briefly, plasmid-transfected MEFs adhered to glass coverslips were supplied with fresh culture medium and incubated for 1 h. *S. aureus* (3×10^5^ cfu) was added to each dish containing MEFs. After 1.5 h incubation at 37°C, the cells were washed three times with culture medium, after which 100 µg/mL gentamicin was added to the culture medium to kill any extracellular *S. aureus*. Infected cells were incubated in culture medium containing 100 µg/mL gentamicin for the duration of the assay.

### Immunostaining and confocal microscopy

To distinguish between intracellular and extracellular *S. aureus*, protein A, a cell wall protein of *S. aureus*, was immunostained under non-permeabilizing conditions, after which 4′,6-diamidino-2-phenylindole (DAPI, Kirkegaard & Perry Laboratories, Inc., Gaithersburg, MD) was used to stain cell nuclei under permeabilizing conditions. *S. aureus* cells stained only with DAPI were identified as intracellularly localized bacteria. Briefly, infected cells were fixed with 4% paraformaldehyde in phosphate buffered saline (PBS) for 10 min, then treated with 50 mM NH_4_Cl/PBS for 10 min, and blocked with 2% bovine serum albumin/PBS for 30 min at room temperature. The cells were then incubated with mouse anti-protein A antibodies (1∶1000) (P2921, Sigma-Aldrich) for 1 h at room temperature. After washing with PBS, the cells were incubated with Alexa 488-conjugated anti-mouse IgG antibodies (1∶1000) for 30 min at room temperature. Cells were permeabilized with 0.2% Triton X-100/PBS for 10 min at room temperature and incubated with 1 µg/mL DAPI/PBS for 1 h at 37°C. To stain autolysosomes, red fluorescent protein (RFP)-LC3-transfected MEFs were infected with green fluorescent protein (GFP)-expressing *S. aureus* (ATCC 29213) for 1.5 h. After washing with culture medium containing 100 µg/mL recombinant lysostaphin (Wako, Osaka, Japan), the cells were further incubated until 3 or 4.5 h post-infection. LysoTracker Blue DND-22 (100 nM, Life Technologies) was added to the medium 15 min before the end of the incubation period. After fixation with 4% paraformaldehyde for 10 min at room temperature, the samples were mounted with PermaFluor aqueous mounting medium (Thermo Fisher Scientific, Runcorn, UK). Images were acquired using a laser scanning confocal microscope (FV10i, Olympus, Tokyo, Japan). For analysis, at least 25 cells were randomly selected from three independent experiments.

### Live-cell imaging

MEFs were grown on glass-bottom dishes (Matsunami Glass, Osaka, Japan) in culture medium without antibiotics. Following bacterial infection, dishes were mounted onto the microscope stage of a fluorescent microscope (BZ-9000, Keyence, Osaka, Japan) equipped with a humidified environment chamber (with 5% CO_2_ at 37°C). Images were acquired every 90 sec using the BZ-9000 microscope equipped with a CFI Plan Apo 10× oil immersion objective (Nikon, Tokyo, Japan), and were processed using the Keyence Bz-II application (Keyence). For the visualization of autophagosomes and autolysosomes, we used the mRFP-GFP-LC3 plasmid, which labels autophagosomes in yellow due to the dual luminescence of RFP and GFP, and autolysosomes in red due to the quenching of green fluorescence in the autolysosomes [31].

### Colony formation assay

Colony formation assays were performed as previously described [8], with some modifications. Briefly, MEFs were cultured to subconfluence in 35-mm diameter dishes, at which point they were supplied with fresh culture medium. *S. aureus* was then added to the culture medium at a multiplicity of infection (MOI) of 100∶1. After 1.5 h, the cells were washed and treated with 100 µg/mL recombinant lysostaphin for 15 min to remove extracellular bacteria, after which the cells were washed three times to remove traces of lysostaphin and dead bacteria. A lysis buffer (PBS containing 0.1% Triton X-100) was added to some culture dishes (1.5 h control samples), while the remaining dishes were incubated until 3 h post-infection before washing and lysis. Lysates were diluted with PBS and spread onto tryptic soy agar plates for colony formation.

### Statistical analysis

Statistical analyses were performed using unpaired two-tailed *t* tests with Welch's correction, or Kruskal-Wallis tests followed by Dunn's multiple comparison tests. A *p-*value of <0.05 was considered statistically significant. Graphs show mean ± standard error of the mean (SEM).

## Results

### *S. aureus* numbers in LC3-positive *S. aureus-*containing autophagosome-like vesicles (SAcVs) are higher in *PRIP*-DKO MEFs

**Figure 1:**
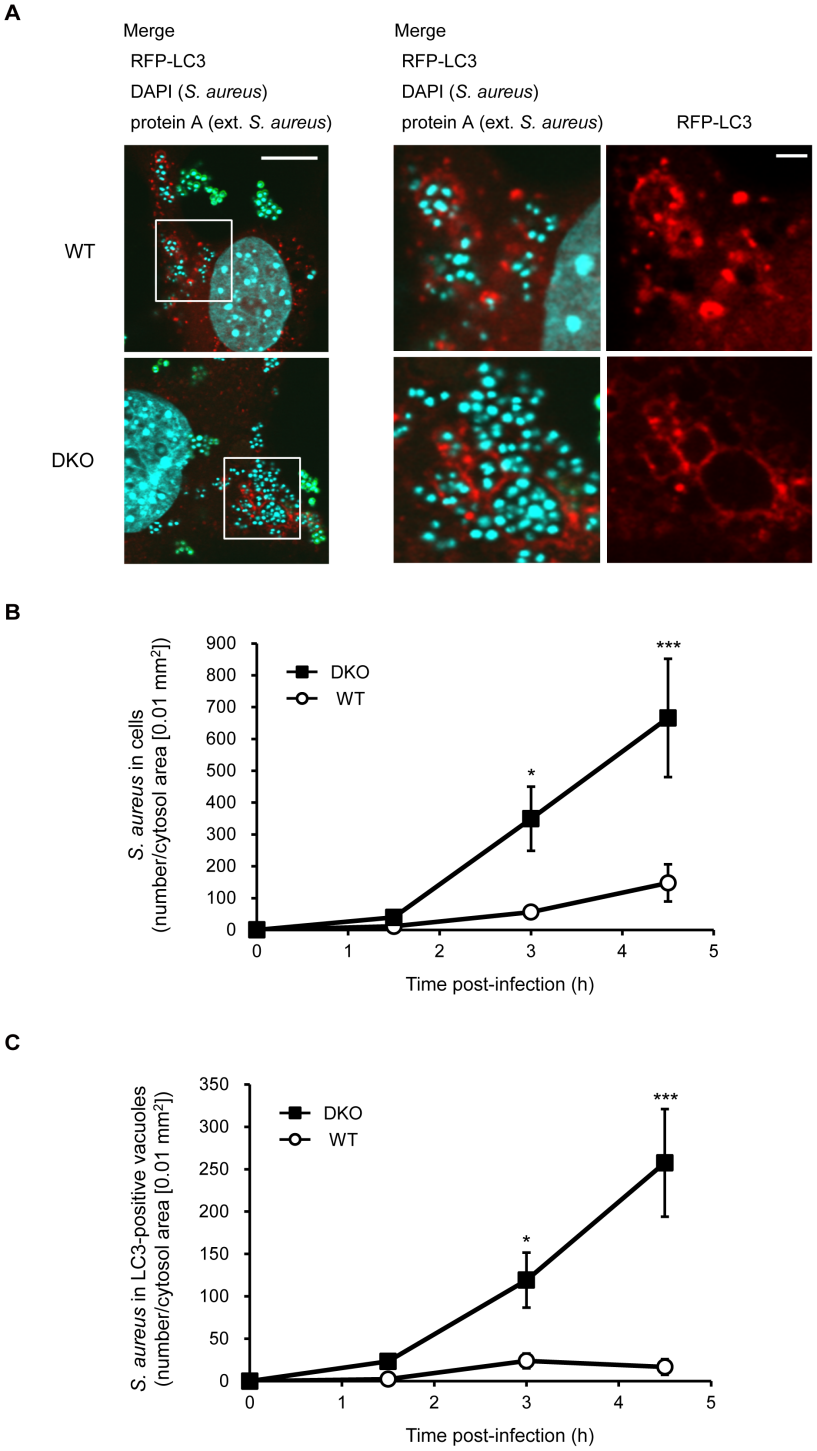
*PRIP* deficiency in mouse embryonic fibroblasts (MEFs) induces *S. aureus* accumulation in autophagosome-like vacuoles. (A–C) Comparison between *S. aureus-*containing autophagosome-like vacuoles in wild-type (WT) and *PRIP* double-knockout (DKO) MEFs. MEFs transiently expressing RFP-LC3 were incubated with *S. aureus* (ATCC 29213). After 1.5 h incubation, cells were washed with 100 µg/mL of gentamicin-containing medium and further incubated with gentamicin-containing medium until 3 or 4.5 h post-infection. Extracellular (ext.) *S. aureus* cells were immunostained with anti-protein A antibody (green), and intracellular *S. aureus* cells were defined as DAPI single-positive signals (A). Images at 3 h post-infection obtained by confocal laser microscopy are shown. Enlarged images of the boxed areas of the left image (scale bar: 10 µm) are shown in the middle and right images (scale bar: 2 µm). Representative images from three independent experiments are shown. The graph in (B and C) shows the numbers of intracellular *S. aureus* cells and of *S. aureus* cells localized to LC3-positive autophagosome-like vacuoles per cytosol area (0.01 mm^2^), respectively. Values represent means ±SEM (n = 20 cells analyzed at each time-point). Reproducible results were obtained from three independent experiments. *p<0.05 and ***p<0.001 relative to corresponding WT values.

PRIP is a newly identified LC3-binding protein that regulates canonical autophagy [2]. Autophagy has recently been highlighted as an important component of the immediate autonomous cell defense mechanism by degrading intracellular pathogens. Therefore, to investigate whether PRIP affects bacterial infection-induced autophagosome-like vacuole formation, mRFP-LC3 transfected MEFs were prepared from WT and *PRIP*-DKO mice and were infected with *S. aureus* ATCC 29213. Cells were then immunostained, followed by confocal microscopic observation. Very few LC3-positive structures surrounding *S. aureus* were observed 1.5 h post-infection (data not shown); however, a large number of LC3-positive SAcVs was observed 3 h after the bacterial infection in WT and *PRIP*-DKO MEFs (Figure 1A).

The number of intracellular bacteria (*S. aureus* assessed by DAPI staining but not by protein A-immunostaining) at 3 h and 4.5 h post-infection was counted. Compared to WT MEFs, the number of *S. aureus* in *PRIP*-DKO MEFs was approximately 6- and 5-fold higher at 3 and 4.5 h post-infection, respectively (Figure 1B). In the WT MEFs, a peak in the number of *S. aureus* entrapped in the LC3-positive vacuoles was at 3 h post-infection (Figure 1C), consistent with the results shown by Schnaith et al. using HeLa cells [8]. On the other hand, in *PRIP*-DKO MEFs, the number of *S. aureus* was robustly increased during the 1.5–4.5 h period post-infection (Figure 1C). The number of *S. aureus* was 5- and 15-fold higher than the control at 3 and 4.5 h post-infection, respectively. Thereby, more cytosol-located bacteria were consistently observed in *PRIP*-DKO MEFs, suggesting that *S. aureus* accumulates (and probably proliferates) in the LC3-positive autophagosome-like vacuoles in *PRIP*-DKO MEFs before escaping into the cytosol.

As observed in canonical autophagy [2], PRIP was co-localized to LC3-positive vacuolar membranes entrapping *S. aureus*, as determined using *S. aureus*-infected WT MEFs transiently transfected with GFP-PRIP and mRFP-LC3 (Figure S1A). Rab7 is a member of the small GTPase Rab family, participating in the formation of pathogen-containing large vacuoles [32] and the fusion step of autophagosomes with lysosomes in canonical autophagy [33], [34]. We therefore investigated the subcellular distribution of *S. aureus* using MEFs transiently transfected with mRFP-LC3 and GFP-Rab7. *S. aureus* cells were more frequently seen to have accumulated in Rab7- and LC3-double-positive SAcVs in *PRIP*-DKO MEFs than in WT MEFs at both 3 and 4.5 h post-infection (Figure S1B–D), suggesting the attenuation of autophagic flux in *PRIP*-DKO MEFs.

### Autophagosomal maturation is suppressed in *PRIP*-DKO MEFs

**Figure 2:**
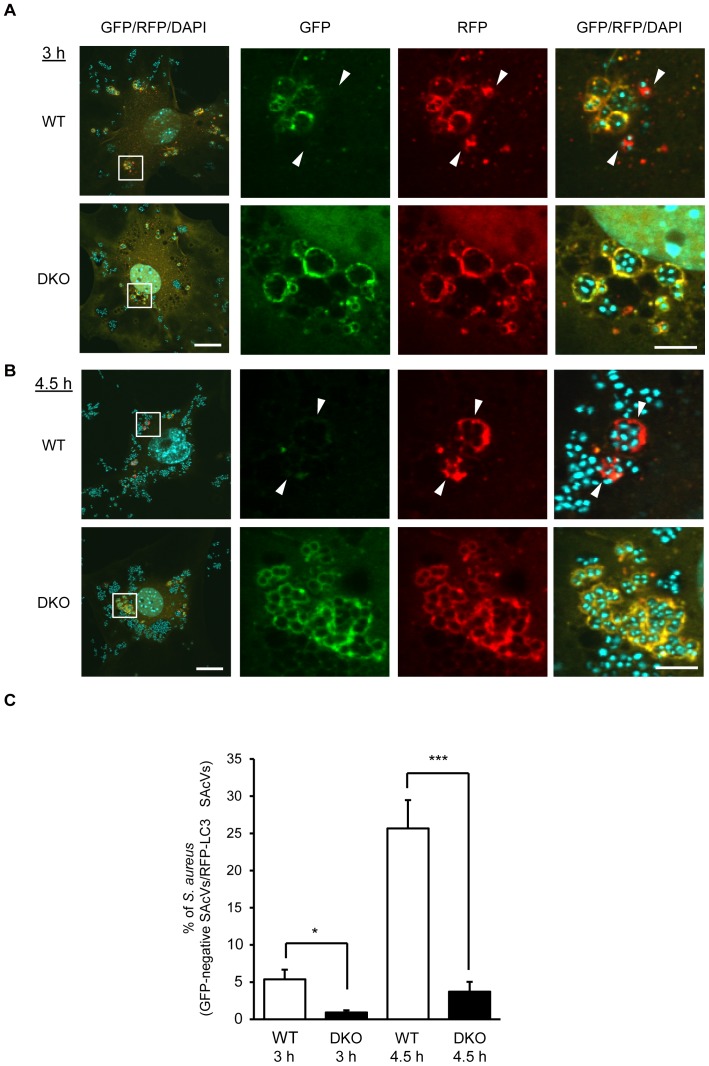
Impairment of autophagosomal maturation in *PRIP*-DKO MEFs. (A, B) MEFs transiently expressing mRFP-GFP-LC3 were incubated with *S. aureus* (ATCC 29213) for 3 h (A) and 4.5 h (B). Images were obtained by confocal laser microscopy. A set of representative images from three independent experiments using wild-type (WT) and *PRIP*-DKO (DKO) MEFs are shown. Bacteria were stained with DAPI (blue). LC3-positive autophagosome-like vacuoles with RFP(+)GFP(−) signal indicate the formation of autolysosomes (arrowheads). Scale bars represent 20 µm in the left panel and 5 µm in the three right panels. (C) The graph shows the ratio of the number of *S. aureus* cells entrapped in RFP(+)GFP(−) vacuoles *vs.* the number of *S. aureus* cells entrapped in RFP(+) vacuoles. Values are expressed as means ±SEM (n = 60 cells for each genotype and each infection time from three independent experiments). WT, 5.4±1.3% (3 h) and 25.7±3.8% (4.5 h); DKO, 0.9±0.3% (3 h) and 3.7±1.3% (4.5 h). *p<0.05, ***p<0.001.

The tandem fluorescent-tagged LC3 (mRFP-GFP-LC3) is a convenient tool to monitor autophagic flux based on the different pH stabilities of the EGFP and mRFP fluorescent proteins. To elucidate the role of PRIP in the autophagy maturation process, MEFs transiently expressing mRFP-GFP-LC3 were infected with *S. aureus*. As shown in Figure 2A, red signals (*i.e.*, green fluorescence-quenched vesicles) appeared in WT MEFs 3 h post-infection, whereas red signals in *PRIP*-DKO cells were infrequently observed. At 3 h post-infection, the ratio of RFP(+)GFP(−) to RFP(+) signals was approximately 5% and <1% in WT and *PRIP*-DKO MEFs, respectively (Figure 2C). This difference was more pronounced at 4.5 h post-infection, where approximately 25% of autophagosome-like vacuoles had been transformed into acidic vesicles (emitting red fluorescence) in WT MEFs, while the proportion of red-emitting vacuoles in *PRIP*-DKO MEFs was as low as 5% (Figure 2B, C), suggesting impairment of autophagic flux. Furthermore, larger RFP(+)GFP(+)-positive vacuoles containing *S. aureus* were observed in *PRIP*-DKO MEFs at 4.5 h post-infection (Figure 2B).

To investigate whether the fusion of autophagosomes with lysosomes in canonical autophagy is also disturbed in *PRIP*-DKO MEFs, an autophagy flux assay was performed using mRFP-GFP-LC3. The number of GFP and RFP-double positive LC3 dots was increased in *PRIP*-DKO MEFs before and after 1 h of nutrient starvation, compared with WT MEFs (Figure S2A), consistent with our previously reported observations [2]. However, the starvation triggered a significant decrease in the number of RFP-single positive LC3 dots in *PRIP*-DKO MEFs than that in WT MEFs, indicating that PRIP positively regulates the fusion of autophagosomes with lysosomes.

### Acidification of autophagosomes is prevented in *S. aureus*-infected *PRIP*-DKO MEFs

**Figure 3:**
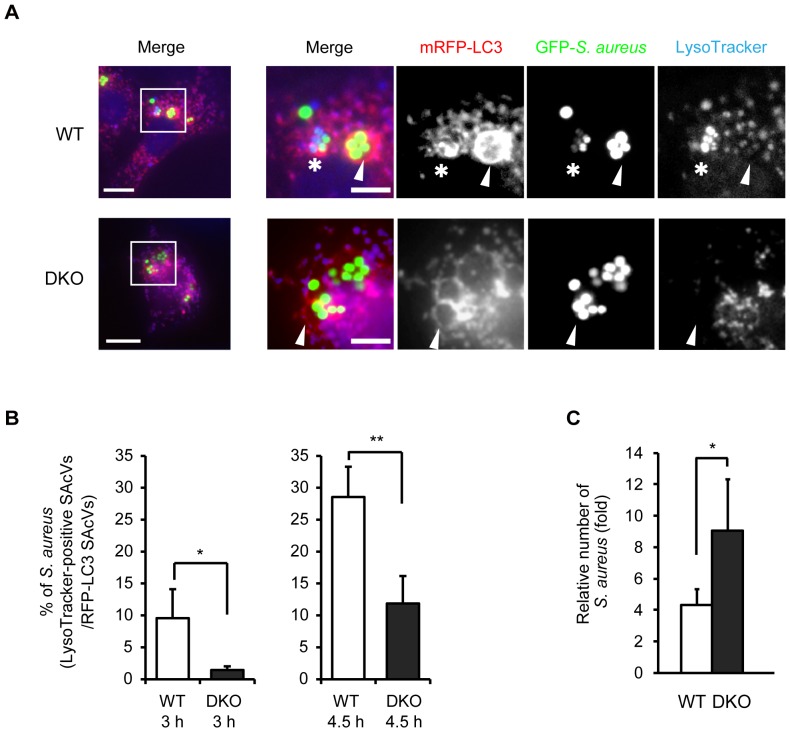
Decreased LysoTracker-positive vacuoles containing *S. aureus* in *PRIP*-DKO MEFs. (A) Co-localization of LC3-positive *S. aureus-*containing autophagosome-like vacuoles (SAcVs) and lysosomal marker (LysoTracker). *PRIP*-DKO (DKO) and wild-type (WT) MEFs transiently expressing mRFP-LC3 were infected with GFP-expressing *S. aureus* (ATCC 29213), after which extracellular bacteria were killed by lysostaphin treatment 1.5 h post-infection. The images were acquired using a fluorescent microscope. Scale bars: 20 µm (left) and 5 µm (right). Asterisks and arrowheads show LC3-positive SAcVs staining with and without LysoTracker, respectively. (B) Comparison of the number of *S. aureus* in LysoTracker-positive vacuoles *vs.* the number of *S. aureus* in mRFP-LC3-positive signals from three independent experiments. WT, 9.6±4.5% (3 h, n = 25) and 28.6±4.7% (4.5 h, n = 28); DKO, 1.4±0.6% (3 h, n = 26) and 11.9±4.3% (4.5 h, n = 25). (C) Colony count assay. Homogenates of *S. aureus*-infected MEFs prepared 1.5 or 3 h post-infection were plated on tryptic soy agar and colony numbers were counted after 16 h incubation at 37°C. Graph represents relative number of colonies (the number of colonies at 3 h *vs.* the number of colonies at 1.5 h). Data represent the means ±SEM (n = 3; WT, 4.3±1.3%; DKO, 9.0±3.3%). *p<0.05, **p<0.01.

To confirm the existence of autolysosome-like acidic vacuoles, we analyzed the acidification of SAcVs in MEFs using LysoTracker blue DND-22, a probe for acidic compartments. LC3-positive SAcVs stained with LysoTracker were observed 3 h post-infection in WT MEFs, but there were fewer apparent in *PRIP*-DKO MEFs (Figure 3A). The acidic compartments were then counted; in WT MEFs, approximately 10% and 30% of LC3-positive SAcVs were stained with LysoTracker at 3 h and 4.5 h post-infection, respectively. However, in *PRIP*-DKO MEFs, there were only about 2% and 10% at 3 h and 4.5 h, respectively (Figure 3B). The acidification process of SAcVs is, therefore, likely attenuated in *PRIP*-DKO MEFs, since there is no quantitative difference in lysosomes between WT and *PRIP*-DKO MEFs before the infection (Figure S2B).

To evaluate the elimination of *S. aureus* in *PRIP*-DKO MEFs at a lower magnitude than that in the control, a colony count assay was performed using cell homogenates of *S. aureus*-infected MEFs collected 1.5 h and 3 h after the infection. No significant difference between the mean numbers of *S. aureus* colonies between WT and *PRIP*-DKO MEFs was observed at 1.5 h post-infection, but the ratio of the colony number at 3 h post-infection relative to that at 1.5 h post-infection was significantly higher in *PRIP*-DKO cells than in WT cells (Figure 3C), suggesting that *S. aureus* infected into *PRIP*-DKO MEFs proliferated in the autophagosomes.

### The lifetime of autophagosome-like vacuoles is prolonged in *PRIP*-DKO MEFs

**Figure 4:**
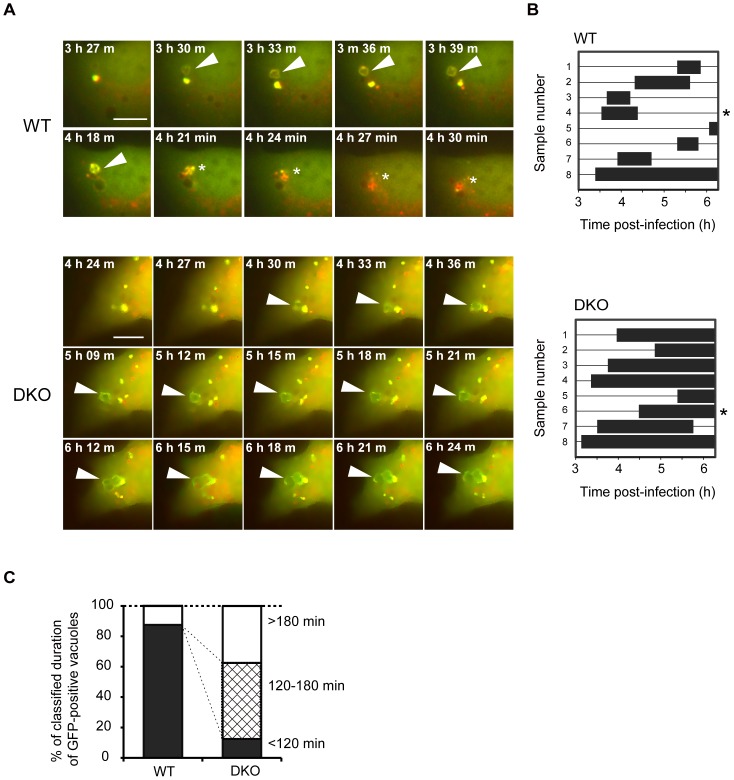
Impairment of autophagic flux in *PRIP-*DKO MEFs. (A–C) Time-lapse analysis of *S. aureus*-infected MEFs. MEFs transiently transfected with the mRFP-GFP-LC3 plasmid were cultured with *S. aureus* (MW2) for 1.5 h, after which extracellular bacteria were killed by lysostaphin treatment. In wild-type (WT) cells (upper panels), a RFP(+)GFP(+)-LC3-labeled autophagosome (arrowhead) appeared at 3 h 30 min post-infection and changed to a RFP(+)GFP(−)-LC3-labeled autolysosome at 4 h 21 min post-infection (asterisk). In *PRIP*-DKO images (DKO, lower panels), an RFP(+)GFP(+)-LC3-labeled autophagosome (arrowhead) appeared at 4 h 30 min post-infection, and the RFP(+)GFP(+) signal (yellow) was not altered during the experiment (terminated at 6 h 24 min post-infection). The closed bars in graphs (B) represent the elapsed time for RFP(+)GFP(+) autophagosome formation in WT (upper) and *PRIP*-DKO (lower) MEFs. Asterisks in (B) indicate the experiment replicates corresponding to images in (A). The graph in (C) shows the proportion of the elapsed times (<120 min: dark shading, 120–180 min: hatched shading, and >180 min: no shading) in WT and *PRIP*-DKO cells.

PRIP appears to positively regulate the fusion process of autophagosomes with lysosomes, and thus, the acidification process would be markedly inhibited in the *PRIP*-DKO MEFs. To monitor the acidification process of SAcVs, we finally performed live-cell imaging using double-tagged LC3 to monitor the fusion of autophagosomes with lysosomes. MEFs were transiently transfected with the mRFP-GFP-LC3 plasmid, followed by infection with *S. aureus* (*agr*-positive strain MW2). As shown in Figure 4A, mRFP-GFP-LC3 appeared as ring-shaped, yellowish structures from about 3 h post-infection in both WT and *PRIP*-DKO genotypes. From 3 h to 6.5 h after the infection in WT cells, newly formed RFP(+)GFP(+) autophagosome-like structures were frequently converted into RFP(+)GFP(−) structures (Movie S1). In *PRIP*-DKO cells, however, rapidly formed yellow-colored autophagosomes rarely changed to red (Movie S2). Using the time lapse movies, we measured how long the yellow-colored period of vesicles lasted, *i.e.*, the time required for change from yellow to red. The mean “yellow periods” were less than 120 min in 90% of WT cells. In *PRIP*-DKO cells, “yellow periods” were significantly longer: in ∼40% and 50% of cells, the periods were >180 min and between 120–180 min, respectively (Figure 4B, C).

## Discussion

Autophagy is not only a cytosolic catabolic process, but also an innate defense mechanism against invading pathogenic bacteria in eukaryotic cells. We have previously reported that PRIP binds LC3, a pivotal component of the autophagic machinery, and is implicated in starvation-induced canonical autophagy [2]. In this study, we demonstrate that PRIP participates in the elimination of *S. aureus* in non-professional phagocytic cells by promoting the acidification of autophagic vacuoles; *i.e*., PRIP promotes the process of the fusion between autophagosomes and lysosomes. To our knowledge, this is the first report on the important role of LC3-mediated autophagic flux via PRIP in host innate immunity against bacterial infection.

In yeast, Atg8 is crucial to the regulation of the autophagic process, specifically in the elongation of the phagophore membrane by mediation of hemifusion events [35], [36]. At least eight mammalian Atg8 orthologs, LC3A, LC3B, LC3B2, LC3C, GABARAP, GABARAPL1 (GEC1), GABARAPL2 (GATE-16), and GABARAPL3 have been identified [37]. The various roles of LC3B have been extensively studied in the process of mammalian autophagy. However, the functions of the other mammalian Agt8 orthologs in autophagy are not fully understood, despite a recent report of the involvement of GABARAP/GATE-16 in autophagosome biogenesis [38]. GABARAP was identified as a PRIP-binding protein by yeast two-hybrid analysis [19], and then we examined that LC3 [2] and GATE-16 (unpublished data) were also able to bind PRIP. In this study, we demonstrated the co-localization of PRIP with LC3 on vacuolar membranes containing *S. aureus* and used LC3 as a tracer protein to visualize autophagosomes and to monitor the autophagy flux in *S. aureus*-infected cells.

Compared with WT MEFs, the number of *S. aureus* cells entrapped in autophagosome-like vesicles was significantly increased in *PRIP*-DKO MEFs, and the *S. aureus* proliferation efficiency was upregulated in these cells. Yamaguchi et al. reported that Rab7 localizes group A streptococcus-containing autophagosome-like vacuoles (GcAV) and mediates the early phase of GcAV formation in NIH3T3 cells [32]. We investigated the co-localization of SAcV with the small GTPase Rab7. Our findings showed that approximately 25% of *S. aureus* entrapped in LC3- and/or Rab7-positive vacuoles was observed in LC3-single positive vacuoles (results not shown), indicating that, unlike GcAV formation, Rab7 may not be essential for the early phase of SAcV formation in MEFs. Therefore, a role for Rab7 in the early phase of SAcV formation is currently unknown. Rab7 is also reported to be directly involved in the fusion process of late endocytic structures with lysosomes [39], and is required for fusion of autophagosomes with lysosomes in the canonical autophagy pathway [33], [34], [40]. Our results showing that more *S. aureus* were entrapped in autophagosome-like vacuoles in *PRIP*-DKO MEFs indicate that PRIP may be participating in the proper formation of autophagosomes in collaboration with Rab7.

To determine whether PRIP may possibly participate in the later steps of autophagy, including fusion between SAcV and lysosome, we performed autophagy flux assays using an mRFP-GFP-tagged LC3 plasmid. The acidification process in LC3-positive SAcVs in the *PRIP*-DKO MEFs was significantly inhibited. Similar effects were observed in autophagy flux assays using another *agr*-positive *S. aureus* strain, MW2. We also elucidated the involvement of PRIP in the starvation-induced autophagosome/lysosome fusion process. Based on these results, we concluded that PRIP participates in the fusion of autophagosomes with lysosomes, and that insufficient autophagosome/lysosome fusion in *PRIP*-DKO MEFs promotes *agr*-positive *S. aureus* replication in autophagosomes, leading to propagation of the bacteria.

The ability of autophagy to either eliminate pathogenic organisms or to provide a niche for their replication depends on the nature of the pathogen [41]. It has been reported that intracellular *agr*-positive, but not *agr*-negative *S. aureus*, becomes sequestered by and replicates in autophagosome-like vesicles following autophagosome/lysosome fusion blockage in HeLa cells [8]. However, it was recently reported that intracellular *agr*-positive *S. aureus* was efficiently entrapped in WIPI-1 positive autophagosome-like vesicles and targeted for lysosomal degradation in non-professional phagocytic cells (human osteosarcoma U2OS cells, ATCC) [9], indicating that *agr*-positive *S. aureus* is eliminated by the autophagy system followed by degradation in the lysosome. The fate of *S. aureus* in a host cell may partly depend on a balance between the ability of the pathogen to escape from autophagosomes and the ability of host cells to eliminate the pathogen by autophagy. In our experiments, approximately 30% of SAcVs in WT MEFs were fused with lysosomes, which is consistent with the previously reported findings of Schnaith et al. (Figure 5B in *Ref*. 8), indicating that *S. aureus* was constitutively eliminated by the autophagosome/lysosome pathway.

WIPI-1-decorated autophagosome-like vacuoles entrapping *S. aureus* induce the degradation of bacteria by lysosomes [9]. WIPI-1 and WIPI-2 are the mammalian orthologs of yeast Atg18, and contain a specific phospholipid-binding region [42]. Moreover, tectonin domain-containing protein 1 (TECPR1) regulates the selective autophagy pathway against *Shigella* in combination with WIPI-2 [43]. TECPR1 has also been shown to be involved in the fusion process of autophagosomes with lysosomes in the canonical autophagy pathway, rigorously mediating the process by associating with both the Atg12-Atg5 conjugate and phosphatidylinositol 3-phosphate [PtdIns(3)P] [44]. Indirect downregulation of PtdIns(3)P levels by *Listeria* phospholipases, phospholipases C A and B, protects *Listeria* from autophagy-mediated clearance [45]. We have shown that PRIP has a pleckstrin homology domain that binds phosphoinositides, and an X-Y phospholipase C catalytic-like domain with no catalytic activity [11], [12]. A recombinant full-length PRIP1 and a recombinant X-Y phospholipase C catalytic-like domain of PRIP were found to be able to bind phosphoinositides including PtdIns(3)P (unreported observation). It is therefore also tempting to speculate that PRIP may regulate autophagy maturation by affecting the functions of TECPR1 through phosphoinositide metabolism in the process of bacterial elimination. Further experiments are needed to fully elucidate the PRIP-regulated autophagosome/lysosome fusion mechanism.

In this study, we show that PRIP is involved in the entrapment of pathogens by LC3-positive autophagosome-like vacuoles and contributes to the autophagic clearance of bacterial pathogens as part of an innate defense system in eukaryotic non-professional phagocytic cells. In *PRIP*-DKO cells, *S. aureus* can escape the host defense system via autophagy due to the autophagosome/lysosome fusion process being disabled. Revealing the functional mechanisms of PRIP-mediated elimination of *S. aureus* from infected host cells gives us new insight into potentially effective treatments for infectious diseases.

## Supporting Information

Figure S1 **Localization of** ***S. aureus*** **entrapped in Rab7 and LC3 double-positive vacuoles.** (A) Co-localization of PRIP with LC3-positive SAcVs. WT-MEFs transiently expressing GFP-PRIP1 and mRFP-LC3 were incubated with *S. aureus* (ATCC 29213) for 3 h. After fixation, bacterial and host DNA were stained with DAPI, and images were obtained by confocal laser microscopy. Enlarged images of the boxed areas of the left image (scale bar: 20 µm) are shown in the right three images (scale bar: 2 µm). Arrowheads indicate representative RFP-LC3-positive vacuoles co-localized with GFP-PRIP signals. *S. aureus* cells were stained with DAPI. More than six similar images were obtained from three independent experiments. (B–D) MEFs transiently transfected with mRFP-LC3 and GFP-Rab7 plasmids were incubated with *S. aureus* (ATCC 29213). After fixation, *S. aureus* cells were stained with DAPI. Images were obtained by confocal laser microscopy. A set of representative images at 3 h (B) and 4.5 h (C) post-infection from four independent experiments are shown. The left images are taken at a low magnification (scale bar: 20 µm), and enlarged images of the boxed areas are shown in the three right images of each set of images (scale bar: 2 µm). The graphs in (D) show the ratio of the number of *S. aureus* in Rab7(+)LC3(+) vacuoles *vs.* the number of *S. aureus* in LC3(+) vacuoles at 3 h and 4.5 h post-infection (left and right panels, respectively). Values are expressed as means ±SEM. [n = 33 (3 h, for each genotype) and n = 24 (4.5 h, for each genotype) cells from three independent experiments]; WT, 44.1±4.0% (3 h) and 41.3±4.8% (4.5 h); DKO, 61.5±4.5% (3 h) and 59.4±4.8% (4.5 h). *p<0.05, **p<0.01.
(TIF)

Figure S2 **Starvation-induced autophagy.** (A) MEFs (WT, DKO) transiently expressing mRFP-GFP-LC3 were cultured on glass coverslips in DMEM containing 10% fetal bovine serum (nutrient-rich) overnight. Then, the medium was replaced with starvation medium, Earle's balanced salt solution, and cells were incubated for 1 h. The cells were fixed with 4% paraformaldehyde for fluorescent microscopy. GFP-positive (yellow bars) and RFP-positive dots were counted, and RFP single-positive dots (red bars) were calculated by subtracting the two values. Values are expressed as means ±SEM [WT and DKO, n = 31 and 31 (0 h); n = 43 and 62 (1 h), respectively]. **p<0.01; n.s., not statistically significant. (B). MEFs were stained with LysoTracker, observed with a fluorescence microscope, and the lysostracker-positive compartments were counted. Scale bar: 20 µm. Values are expressed as means ±SEM (WT and DKO, n = 36 and 36, respectively). n.s., not statistically significant.
(TIF)

Movie S1 **Time-lapse analysis of** ***S. aureus*** **-infected WT MEFs.** Time-lapse analysis of *S. aureus* (MW2 strain)-infected WT-MEFs transiently transfected with the mRFP-GFP-conjugated LC3 plasmid. Images were captured every 90 s from 3 h to 6 h 15 min post-infection. Movie corresponds to Figure 4A (WT).
(MOV)

Movie S2 **Time-lapse analysis of** ***S. aureus*** **-infected** ***PRIP*** **-DKO MEFs.** Time-lapse analysis of *S. aureus* (MW2 strain)-infected *PRIP* double-knockout (*PRIP*-DKO) mouse embryonic fibroblasts (MEFs) transiently transfected with the mRFP-GFP-conjugated LC3 plasmid. Images were captured every 90 s from 3 h to 6 h 27 min post-infection. Movie corresponds to Figure 4A (DKO).
(MOV)
