# Supplementary figures and images for: Phospholipase C-Related Catalytically Inactive Protein Participates in the Autophagic Elimination of Staphylococcus aureus Infecting Mouse Embryonic Fibroblasts
Source: PLoS One. 2014 May 27;9(5):e98285. doi: 10.1371/journal.pone.0098285 (PMC4035314; doi:10.1371/journal.pone.0098285)

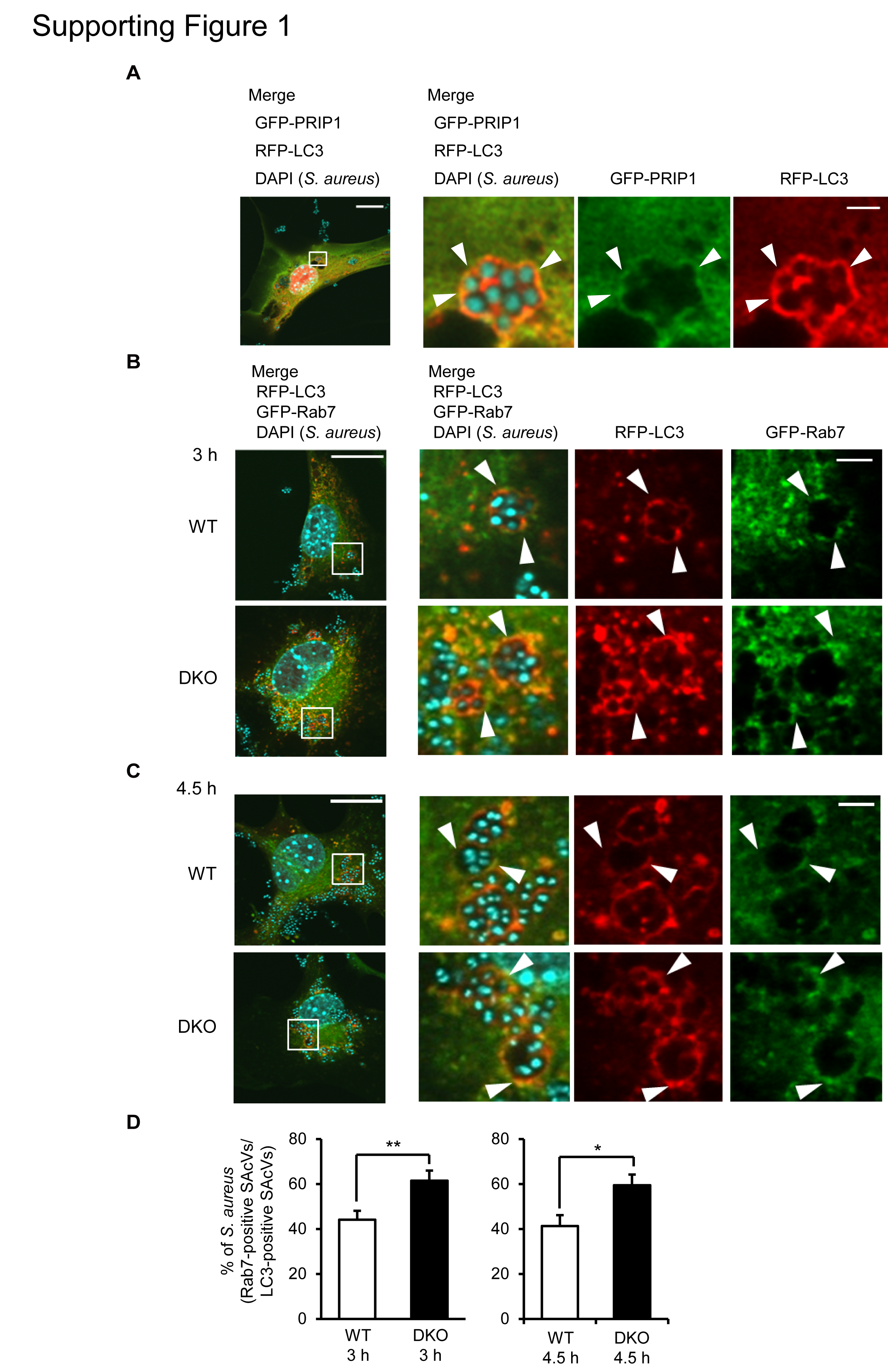

Supplement: Figure S1 — Localization of S. aureus entrapped in Rab7 and LC3 double-positive vacuoles. (A) Co-localization of PRIP with LC3-positive SAcVs. WT-MEFs transiently expressing GFP-PRIP1 and mRFP-LC3 were incubated with S. aureus (ATCC 29213) for 3 h. After fixation, bacterial and host DNA were stained with DAPI, and images were obtained by confocal laser microscopy. Enlarged images of the boxed areas of the left image (scale bar: 20 µm) are shown in the right three images (scale bar: 2 µm). Arrowheads indicate representative RFP-LC3-positive vacuoles co-localized with GFP-PRIP signals. S. aureus cells were stained with DAPI. More than six similar images were obtained from three independent experiments. (B–D) MEFs transiently transfected with mRFP-LC3 and GFP-Rab7 plasmids were incubated with S. aureus (ATCC 29213). After fixation, S. aureus cells were stained with DAPI. Images were obtained by confocal laser microscopy. A set of representative images at 3 h (B) and 4.5 h (C) post-infection from four independent experiments are shown. The left images are taken at a low magnification (scale bar: 20 µm), and enlarged images of the boxed areas are shown in the three right images of each set of images (scale bar: 2 µm). The graphs in (D) show the ratio of the number of S. aureus in Rab7(+)LC3(+) vacuoles vs. the number of S. aureus in LC3(+) vacuoles at 3 h and 4.5 h post-infection (left and right panels, respectively). Values are expressed as means ±SEM. [n = 33 (3 h, for each genotype) and n = 24 (4.5 h, for each genotype) cells from three independent experiments]; WT, 44.1±4.0% (3 h) and 41.3±4.8% (4.5 h); DKO, 61.5±4.5% (3 h) and 59.4±4.8% (4.5 h). *p<0.05, **p<0.01. (TIF) [file pone.0098285.s001.tif]

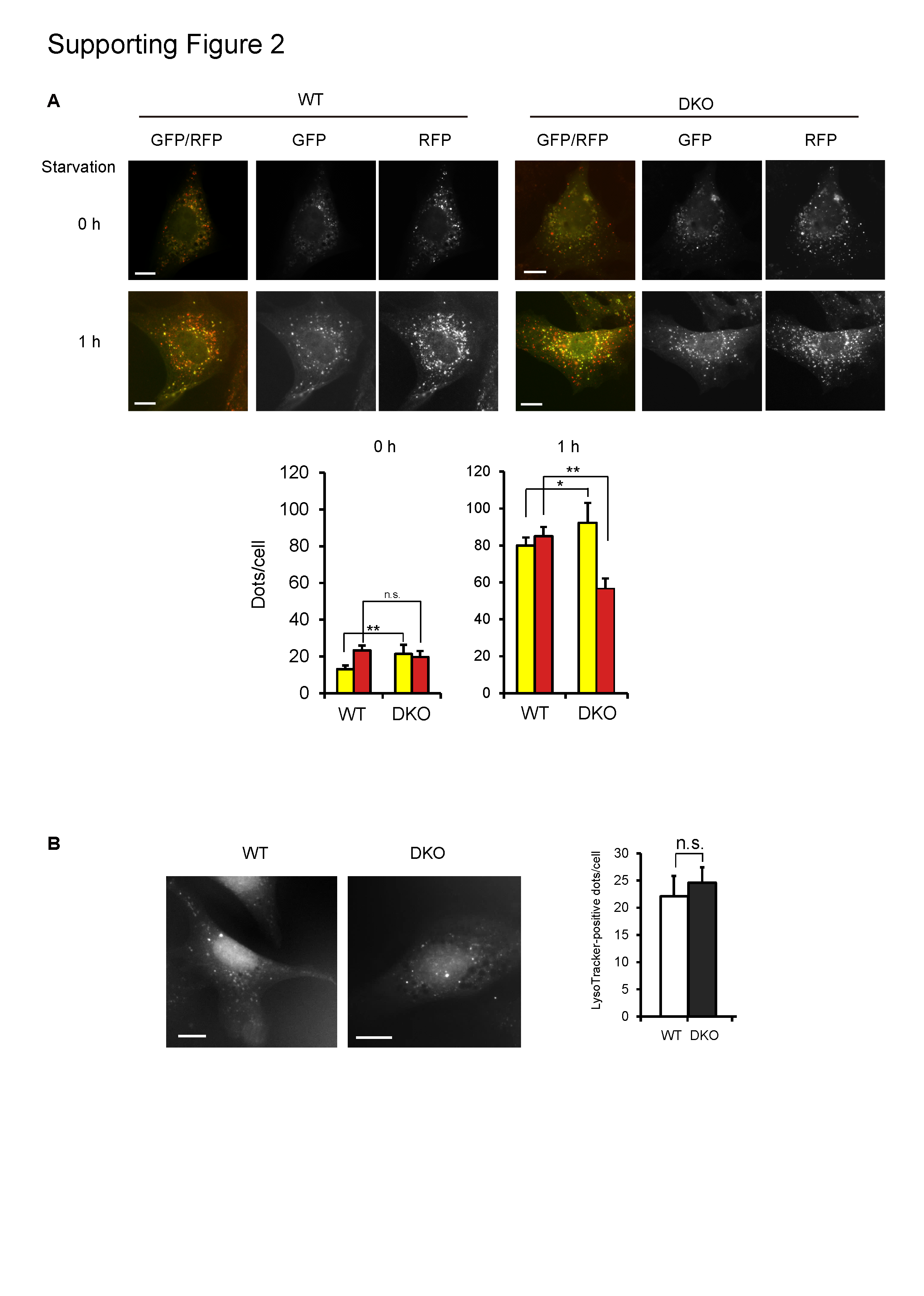

Supplement: Figure S2 — Starvation-induced autophagy. (A) MEFs (WT, DKO) transiently expressing mRFP-GFP-LC3 were cultured on glass coverslips in DMEM containing 10% fetal bovine serum (nutrient-rich) overnight. Then, the medium was replaced with starvation medium, Earle's balanced salt solution, and cells were incubated for 1 h. The cells were fixed with 4% paraformaldehyde for fluorescent microscopy. GFP-positive (yellow bars) and RFP-positive dots were counted, and RFP single-positive dots (red bars) were calculated by subtracting the two values. Values are expressed as means ±SEM [WT and DKO, n = 31 and 31 (0 h); n = 43 and 62 (1 h), respectively]. **p<0.01; n.s., not statistically significant. (B). MEFs were stained with LysoTracker, observed with a fluorescence microscope, and the lysostracker-positive compartments were counted. Scale bar: 20 µm. Values are expressed as means ±SEM (WT and DKO, n = 36 and 36, respectively). n.s., not statistically significant. (TIF) [file pone.0098285.s002.tif]
